# The Persian Translation and validation of the celiac disease quality of life questionnaire (CDQOL)

**DOI:** 10.1186/s12955-021-01694-z

**Published:** 2021-02-10

**Authors:** Zeinab Nikniaz, Mohammad Asghari Jafarabadi, Saeedeh Ghaffarifar, Zahra Ravand, Zahra Akbari Namvar, Masood Shirmohammadi

**Affiliations:** 1 Liver and Gastrointestinal Diseases Research Center, Tabriz University of Medical Sciences, Tabriz, Iran; 2 Road Traffic Injury Research Center, Department of Epidemiology and Biostatistics, Faculty of Health, Tabriz University of Medical Sciences, Tabriz, Iran; 3 Medical Education Research Centre, Health Management and Safety Promotion Research Institute, Tabriz University of Medical Sciences, Tabriz, Iran; 4 Student Research Committee, Tabriz University of Medical Sciences, Tabriz, Iran

**Keywords:** Celiac disease, CDQOL, Persian, Validation

## Abstract

**Background:**

Considering the importance of having a celiac disease-specific measure of the quality of life (QOL) in Persian, the present study aimed to translate the celiac disease quality of life questionnaire (CDQOL) into Persian and evaluate its psychometric properties.

**Methods:**

In this cross-sectional study, the Forward–Backward translation method was used. The content validation ratio (CVR) and the content validity index (CVI) were used for content validity assessment. The construct validity was assessed by exploratory factor analysis (EFA) and confirmatory factor analysis (CFA) on 220 celiac patients who were selected randomly from the celiac disease (CD) registry database. The correlations between the result of the Persian version of CDQOL (PCDQOL), self-rated QOL, and short form-36 (SF36) were analyzed using the Pearson correlation test. The internal consistency and test–retest reliability were measured through Cronbach’s alpha and intraclass correlation coefficient (ICC).

**Results:**

In the present study, 220 celiac patients with a mean age of 35.54 ± 10.29 years participated. The mean CVI, CVR, and impact score of PCDQOL were 0.98, 0.96, and 4.82 respectively. Using EFA, four factors have extracted that had a good fit in CFA (Chi-square/DF = 1.74, RMSEA: 0.08, and CFI: 0.90, and NFI: 0.90). The results showed that there was a moderate to high correlation between PCDQOL, SF36 (r: 0.587, *p* = 0.02), and self-rated QOL (r: 0.64, *p* < 0.001). The questionnaire had high internal consistency (Cronbach alpha: 0.93) and test–retest reliability (ICC: 0.96 [0.86–0.99]).

**Conclusion:**

The PCDQOL questionnaire could be used by physicians and nutritionists to assess HRQOL in celiac patients in Iran.

## Background

Celiac disease (CD) is an immune reaction to eating gluten in people with genetic susceptibility. According to the result of a recent systematic review, CD is a common disease with a prevalence of 0.7 to 1% [1]. Its prevalence in Iran is also similar to that of the American and European populations [2]. A strict, life-long gluten-free diet (GFD) is the only available treatment for CD [3]. In this diet, all gluten-containing foods should be eliminated from the diet. Considering that gluten is found in a wide range of foods, adherence to GFD is challenging [4]. On the other hand, the CD could negatively affect the quality of life (QOL) of patients and some studies showed that adherence to GFD could improve the QOL in this population [5]. In clinical studies, improvement in health-related quality of life (HRQOL) in patients with chronic diseases such as celiac is considered a primary outcome [6]. So, its precise measuring is important for determining the efficacy of therapeutic interventions. In this regard, different disease-specific questionnaires were developed and validated for assessing HRQOL in adult celiac patients such as the celiac disease questionnaire (CDQ) [7] and celiac disease quality of life (CDQOL) [8]. The CDQ questionnaire is mostly focused on symptoms and diminishing of daily function and the Persian version is available [9]. However, CDQOL is focused on the problems of everyday life of celiac patients, their limitations, and expectations. The original questionnaire of CDQOL was in English and consists of 20 questions and has been translated into different languages such as Spanish [10], Italian [11], and Dutch [12]. To the best of our knowledge, there has not been any Persian version. Considering the importance of having a celiac disease-specific measure of the quality of life in Persian, the present study aimed to translate CDQOL into Persian and evaluate the psychometric properties of the Persian version.

## Methods

In the present explorative cross-sectional study, the English version of the CDQOL questionnaire was translated to Persian and its face, content, and construct validities as well as its reliability, was examined.

The Forward–Backward translation method was used for the translation of the questionnaire. For this, at first, the permission of the Mapi-Research-Trust was taken by Email. Then two translators who were fluent in English independently translated the questionnaire into Persian (forward translation). The two versions were compared and any inconsistencies were resolved by discussion. This version was back-translated to English by an independent bilingual speaker who was unaware of the English version. This version of the questionnaire was sent to the representative of the Mapi-Research-Trust to confirm.

### Statistical analysis

We analyzed the data using the IBM© SPSS© Statistics version 20 and IBM SPSS AMOS version 26.0.0.

### Assessment of face validity

Face validity was assessed by the panel of experts (11 experts) and oral feedback of 10 celiac patients immediately after the completion of the questionnaire. The experts were asked to provide their opinion about the importance of each question on the 5 points Likert scale. The impact score was calculated and the values greater than 1.5 were considered acceptable. According to patients and expert opinion, some changes in questions number 3 and 17 (adding synonyms of words) were made and the final Persian version of CDQOL (PCDQOL) was created.

### Assessment of content validity

The PCDQOL questionnaire was tested for content validity in a group of five gastroenterologists and four psychologists and two nutritionists. The experts were asked to complete a form to evaluate the simplicity, clarity, relevance, and necessity of each question. We computed the content validation ratio (CVR) for each item based on the Lawshe method [13]. The minimum acceptable level of CVR was 0.59. The content validity index (CVI) was also calculated for each item and the minimum acceptable agreement score applied in this study was 0.76.

### Assessment of construct validity

The construct validity of the questionnaire was assessed in two groups of celiac patients using factor analysis.

For assessing the construct validity, the sampling population was the celiac patients who were registered in the East-Azerbaijan celiac disease registry database. The patients were selected randomly using a computer-generated random number. For all patients, the diagnosis of celiac disease was based on abnormal duodenal histology and positive serology. The inclusion criteria were age 18–70 years, not being pregnant, the absence of major psychiatric disorders, and registered in the East Azerbaijan celiac disease registry database. The sample size was calculated based on the Gorsuch recommendation, with the N:P ratio of 5. In this ratio “N” is the minimum sample size and “P” is the number of questions [14]. As in the present study, “P” was 20, we needed a sample size of 100 participants at least. A total of 220 celiac patients, were recruited. Participants were randomly divided into two groups. EFA was conducted on 120 patients, and CFA was performed on 100 patients. The data collected included age, sex, age at diagnosis, family history of celiac disease.

The patients were asked to complete the PCDQOL questionnaire. This questionnaire has 20 questions on the five liker scale. The scores are summed to obtain a final score (from 20 to 100). A higher score indicating a better quality of life.

Exploratory factor analysis (EFA) was done according to the original article method through principal axis analysis followed by a varimax rotation to test the factor constructs of all the 20 items. For confirming the sample adequacy the Kaiser–Meyer–Olkin (KMO) test and Bartlett’s test of sphericity were used. To check the normality, we applied the statistical method of Skewness and Kurtosis. Skewness within ± 1.5 and kurtosis within ± 2 was considered as an indication of normality [15].

Confirmatory factor analysis (CFA) was conducted to assess the goodness of fit between a hypothesized model provided in the original paper and the data obtained from 100 celiac patients in the present study who did not participate in the EFA. Chi-square, root mean error of approximation (RMSEA), comparative fit index (CFI), and normed fit index (NFI) were measured. The acceptable values were Chi-square *p* value > 0.05, RMSEA < 0.08, CFI & NFI > 0.9.

### Assessment of convergent validity

The convergent validity was assessed by analyzing the correlation between the result of PCDQOL, self-rated QOL, and short form-36 (SF36) using the Pearson correlation test.

Self-rated QOL is a single question in which the patients rate their overall QOL from poor to excellent.

SF-36 is a 36-item questionnaire that assesses physical and psychological health with a higher score indicating better health [16].

### Reliability assessment

The internal consistency was measured through Cronbach’s alpha. Besides, the ceiling and flooring effect is measured and values lower than 20% were considered acceptable. For calculating internal consistency, the mean scores of 120 questionnaires were used.

Test–retest reliability was assessed on 20 random samples of patients. The patients were asked to complete the questionnaire two weeks apart. The intraclass correlation coefficient (ICC) was used for analysis.

The present study was approved by the Ethics Committee of Tabriz University of medical sciences (IR.TBZMED.REC.1398.193). Written informed consent was obtained from all participants.

## Results

In the present study, 220 celiac patients participated in the construct validity of PCDQOL. The baseline characteristics of the participants are provided in Table 1. The mean of participants’ age was 35.54 ± 10.29 years and 57.35% of them were female. The mean disease duration was 6.05 ± 7.44 years.

**Table 1:** Baseline characteristics of celiac patients who participated in psychometrics properties assessment (n = 220)

Variable	Mean ± SD
Age (years)	35.54 ± 1.29
Disease duration (years)	6.26 ± 7.44

	Frequency (%)
Sex (M:F)	94 (42.72)/126 (57.27)
Married	164 (74.50)
Positive family history of celiac	34 (15.45)
Education level	
Illiterate	39 (17.72)
High school	149 (67.72)
University	32 (14.55)

M, male; F, female

The face and content validity scores of the questionnaire were shown in Table 2. The mean CVI, CVR, and impact score of PCDQOL were 0.98, 0.96, and 4.82 respectively.

**Table 2:** The results for the normality test, content and face validity of Persian version of CDQOL questionnaire

Items	CVI*	CVR**	IS***
1. I feel limited by this disease	0.97	1	4.45
2. I feel worried that I will suffer from this disease	0.94	0.82	4.27
3. I feel concerned that this disease will cause other health problems	1	1	4.91
4. I feel worried about my increased risk of cancer from this disease	1	1	4.91
5. I feel worried about my increased risk of cancer from this disease	0.94	0.82	4.64
6. I feel like I'm limited in eating meals with coworkers	1	1	4.91
7. I feel like I am not able to have special foods like birthday cake and pizza	1	1	4.91
8. I feel that the diet is sufficient treatment for my disease	1	1	4.92
9. I feel that there are not enough choices for treatment	0.94	1	4.55
10. I feel depressed because of my disease	1	1	5
11. I feel frightened by having this disease	1	1	5
12. I feel like I don't know enough about the disease	1	0.82	5
13. I feel overwhelmed about having this disease	0.87	0.82	4.58
14. I have trouble socializing because of my disease	0.97	1	4.92
15. I find it difficult to travel or take long trips because of my disease	1	1	5
16. I feel like I cannot live a normal life because of my disease	1	1	5
17. I feel afraid to eat out because my food may be contaminated	1	1	5
18. I feel worried about the increased risk of one of my family members having celiac disease	1	1	5
19. I feel like I think about food all the time	1	1	4.83
20. I feel concerned that my long term health will be affected	1	1	5

CVI, content validity index; CVR, content validity ratio; IS, Impact Score

*values > 0.59 are acceptable; ** values > 0.79 are acceptable; *** values > 1.5 are acceptable

Table 3 shows the results of the factor analysis. As can be seen, four factors have extracted that account for 60% of the variance. The results of KMO and Barlett sphericity test**s** showed the adequacy of sample size (KMO: 0.90 and Bartlett's Test of Sphericity p-value < 0.001). The extracted factors were named according to the original paper as following: limitations, dysphoria, health concerns, and inadequate treatment.

**Table 3:** Results of Exploratory Factor analysis of PCDQOL (n = 120)

Items	Skewness	Kurtosis	Factor 1 Limitation	Factor 2 dysphoria	Factor 3 Health concerns	Factor 4 Inadequate treatment
Q 15. I find it difficult to travel or take long trips because of my disease	− 0.1	− 1.44	0.75	–	–	
Q 6. I feel like I'm limited in eating meals with coworkers	− 0.2	− 1.4	0.71	–	–	
Q 7. I feel like I am not able to have special foods like birthday cake and pizza	− 0.1	− 1.33	0.69	–	–	
Q 16. I feel like I cannot live a normal life because of my disease	0.43	− 1.08	0.63	–	–	
Q 19. I feel like I think about food all the time	0.05	− 1.38	0.63	–	–	
Q 14. I have trouble socializing because of my disease	0.77	− 0.85	0.60	–	–	
Q 1. I feel limited by this disease	0.09	− 1.02	0.54	–	–	
Q 5. I feel worried about my increased risk of cancer from this disease	0.6	− 0.06	0.50	–	–	
Q 17. I feel afraid to eat out because my food may be contaminated	− 0.41	− 1.13	0.50	–	–	
Q 13. I feel overwhelmed about having this disease	0.72	− 0.79	–	0.75	–	
Q 11. I feel frightened by having this disease	0.52	− 1.09	–	0.73	–	
Q 10. I feel depressed because of my disease	0.62	− 1.04	–	0.70	–	
Q 12. I feel like I don't know enough about the disease	0.68	− 0.52	–	0.55	–	
Q 3. I feel concerned that this disease will cause other health problems	− 0.2	− 1.2	–	–	0.77	
Q 2. I feel worried that I will suffer from this disease	− 0.04	− 1.2	–	–	0.69	
Q 4. I feel worried about my increased risk of cancer from this disease	0.09	− 1.32	–	–	0.68	
Q 18. I feel worried about the increased risk of one of my family members having celiac disease	− 0.55	− 1.17	–	–	0.55	
Q 9. I feel that there are not enough choices for treatment	0.12	− 1.00	–	–	0.54	
Q 8. I feel that the diet is sufficient treatment for my disease	− 0.30	− 1.26	–	–	–	0.54
Q 20. I feel concerned that my long term health will be affected	− 0.33	− 1.13	–	–		0.54

Extraction method: Principal Axis Factoring. Rotation method: Varimax with Kaiser Normalization

**Fig. 1:**
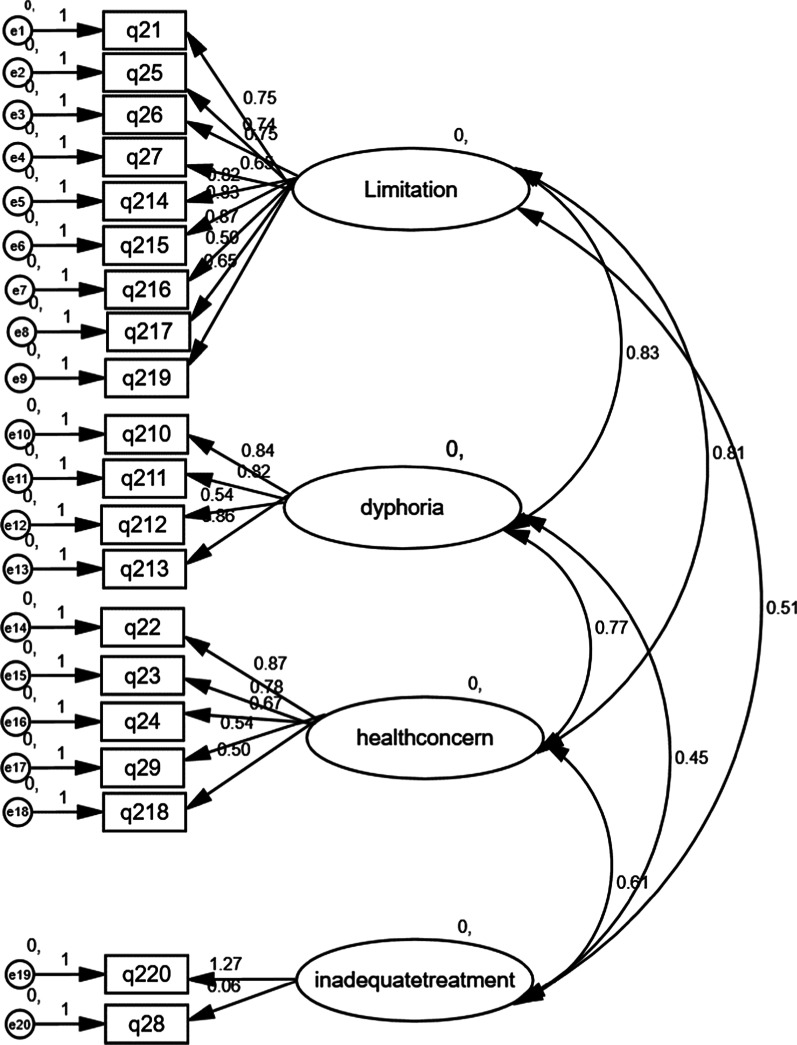
Results of confirmatory factor analysis

The results of the confirmatory factor analysis and goodness of fit indicators are provided in Fig. 1 and Table 4 respectively. According to these results, the four-factor model had a good fit in the Persian data (Chi-square/DF = 1.74, RMSEA: 0.08, and CFI: 0.90, and NFI: 0.90).

**Table 4:** Results of Confirmatory Factor analysis of PCDQOL (n = 100)

Fit statistic	Values
Chi-square/DF	1.74
Root mean squared error of approximation (RMSEA)	0.08
Comparative fit index (CFI)	0.90
Normed fit index (NFI)	0.80

DF, degree of freedom

At the stage of determining convergent validity, the correlations between CDQOL total score and SF36 total score, and self-rated QOL was assessed and the results showed that there was a moderate to high correlation between CDQOL and SF36 (r: 0.587, *p* = 0.02), and self-rated QOL (R:0.64, *p* < 0.001) in the expected direction.

For reliability assessment, the Floor and Ceiling effects were calculated that were 1.6% and 0.8% respectively. The Cronbach-alpha of overall CDQOL score, limitations, dysphoria, and health concerns were 0.93, 0.91, 0.85, and 0.86 respectively. The inadequate treatment scale was identified only by two items, therefore we did not calculate Cronbach-alpha for these factors. The results of the ICC analysis indicated that the questionnaire had high test–retest reliability (ICC: 0.96 [0.86–0.99]).

## Discussion

Different disease-specific questionnaires have been developed and validated to assess the health-related quality of life in patients with celiac disease. In this regard, CDQOL is a valid questionnaire that was developed to assess HRQOL that has clinical relevance [8]. This questionnaire was translated and validated in different languages. However, as far as we know, no Persian version is available. So, in the present study, we translated this questionnaire into Persian and evaluated the psychometric properties in celiac patients. According to the results, the PCDQOL had good content validity. In the factor analysis model, four factors were extracted that confirms the structure of CD-QOL in the number of factors. The original questionnaire also had four factors. The first factor (limitations) includes nine items, the second one (dysphoria) includes four items, the third one (health concerns) includes five items, and the last factor (inadequate treatments) includes two items. This supports the construct validity of this questionnaire. In the combination of items, two factors (limitations and dysphoria) are the same as the original English version. However, in the Persian version, question 9 was loaded on factor 3 (health concerns) instead of factor 4 in the original version, and question 20 was loaded on factor 4 (inadequate treatments) instead of factor 3 in the original version. In the Italian version, Zingone et al. also reported some discrepancies in the combination of items but not in the number of factors [11]. These inconsistencies between the results of different studies may be due to the differences in characteristics of the studied populations such as age and race. Besides, as suggested by Zongone et al., the availability of medical care can affect these results [16]. Besides, in Iran, subvention is given to patients to buy essential gluten-free products such as bread and floor that may affect the results.

For assessing convergent validity, the correlation between the results of the PCDQOL, SF-36, and self-rated QOL was studied and the results indicated that these tests were moderate to highly correlated. The English version had also a moderate to high correlation with IBS-QOL and self-rated QOL [8]. The Italian version had also high correlation with SF-36 (r = 0.55, *p* < 0.001) and self-rated QOL (r = 0.62, *p* < 0.001) [11].

The results of reliability tests showed that the PCDQOL had high internal consistency and test–retest reliability. The original English version of the questionnaire had also high internal consistency (Cronbach-alpha > 0.7). Previous studies also showed the high Cronbach-alpha for Spanish (Cronbach-alpha: 0.9), Italian (Cronbach-alpha = 0.88), and Dutch (Cronbach-alpha: 0.91) versions [10–12]. In line with these results, the PCDQOL had Cronbach-alpha of 0.93 that indicated the high internal consistency.

The present study suffers from some limitations. We just tested the questionnaire on celiac patients in East-Azerbaijan-Iran. This may normally restrict the generalization of its results for other populations and cultures. However, we selected patients randomly from the East-Azerbaijan registry database. So, the sample could better represent East-Azerbaijan celiac patients. Moreover, the sample size was limited. So, it is suggested that future studies evaluate the validity of the questionnaire in other populations with larger sample size.

## Conclusions

In conclusion, according to the results, the PCDQOL questionnaire had high validity, reliability, and internal consistency. So it could be used by physicians and nutritionists to assess HRQOL in celiac patients in Iran.

## Data Availability

The datasets supporting the conclusions of this research are included within the article.

## Abbreviations

CD: Celiac disease
CDQ: Celiac disease questionnaire
CDQOL: Celiac disease quality of life
CFA: Confirmatory factor analysis
CFI: Comparative fit index
CVI: Content validation index
CVR: Content validity ratio
EFA: Exploratory factor analysis
GFD: Gluten free diet
HRQOL: Health related quality of life
ICC: Intraclass correlation coefficient
KMO: Kaiser–Meyer–Olkin
NFI: Normed fit index
PCDQOL: Persian version of CDQOL
QOL: Quality of life
RMSEA: Root mean error of approximation
SF-36: Short-form-36
